# Psychosocial Impact of COVID-19 Nursing Home Restrictions on Visitors of Residents With Cognitive Impairment: A Cross-Sectional Study as Part of the Engaging Remotely in Care (ERiC) Project

**DOI:** 10.3389/fpsyt.2020.585373

**Published:** 2020-10-26

**Authors:** Rónán O'Caoimh, Mark R. O'Donovan, Margaret P. Monahan, Caroline Dalton O'Connor, Catherine Buckley, Caroline Kilty, Serena Fitzgerald, Irene Hartigan, Nicola Cornally

**Affiliations:** ^1^Department of Geriatric Medicine, Mercy University Hospital, Cork, Ireland; ^2^Catherine McAuley School of Nursing and Midwifery, University College Cork, Cork, Ireland; ^3^Northridge House Education and Research Centre, St. Lukes Home, Cork, Ireland

**Keywords:** COVID-19, cognitive impairment (CI), nursing homes (source: MeSH), psychological well-being, Loneliness (source: MeSH, NLM)

## Abstract

**Background:** COVID-19 has disproportionately affected older people. Visiting restrictions introduced since the start of the pandemic in residential care facilities (RCFs) may impact negatively on visitors including close family, friends, and guardians. We examined the effects of COVID-19 visiting restrictions on measures of perceived loneliness, well-being, and carer quality of life (QoL) amongst visitors of residents with and without cognitive impairment (CI) in Irish RCFs.

**Methods:** We created a cross-sectional online survey. Loneliness was measured with the UCLA brief loneliness scale, psychological well-being with the WHO-5 Well-being Index and carer QoL with the Adult Carer QoL Questionnaire (support for caring subscale). Satisfaction with care (“increased/same” and “decreased”) was measured. A history of CI was reported by respondents. Sampling was by convenience with the link circulated through university mail lists and targeted social media accounts for 2 weeks in June 2020.

**Results:** In all, 225 responses were included of which 202 noted whether residents had reported CI. Most of the 202 identified themselves as immediate family (91%) and as female (82%). The majority (67%) were aged between 45 and 64 years. Most (80%) reported that their resident had CI. Approximately one-third indicated reduced satisfaction (27%) or that restrictions had impaired communication with nursing home staff (38%). Median loneliness scores were 4/9, well-being scores 60/100 and carer QoL scores 10/15. Visitors of those with CI reported significantly lower well-being (*p* = 0.006) but no difference in loneliness (*p* = 0.114) or QoL (*p* = 0.305). Reported CI (*p* = 0.04) remained an independent predictors of lower WHO-5 scores, after adjusting for age, sex, RCF location, and dementia stage (advanced), satisfaction with care (reduced), and perception of staff support measured on the Adult Carer QoL Questionnaire.

**Conclusion:** This survey suggests that many RCF visitors experienced low psychosocial and emotional well-being during the COVID-19 lockdown. Visitors of residents with CI report significantly poorer well-being as measured by the WHO-5 than those without. Additional research is required to understand the importance of disrupted caregiving roles resulting from visiting restrictions on well-being, particularly on visitors of residents with CI and how RCFs and their staff can support visitors to mitigate these.

## Background

Coronavirus disease 2019 (COVID-19) has disproportionately affected older adults (1), including residents in nursing homes (2). To date, over 40% of total confirmed COVID-19 deaths have occurred in Residential Care Facilities (RCF) (3). Residents are at increased risk of COVID-19 infection and experience more complications (3). To curb transmission, guidance on strict public health measures have been issued in many countries including restrictions on visiting nursing homes (4, 5).

COVID-19 has also had a negative impact on people with dementia (6) including those in RCF (7). International experts and societies such as Alzheimer's Disease International recommend health authorities provide integrated, interdisciplinary, and collaborative support to people with dementia and their caregivers (8). This may reduce the risk of compromised care and reductions in quality of life (QoL) during this challenging time (8). The psychological effects of COVID-19 broadly and specifically on vulnerable groups such as people with dementia and their caregivers are poorly studied. The need for such research is pressing and supported by mental health advocates including the UK Academy of Medical Sciences (9).

Visits from family and friends are central to the care of residents, buffering against loneliness, anxiety, and depression by providing continuity, advocacy, and emotional support. Visitors (family members and friends) also assist with personal care (10, 11). Visiting can provide residents with a sense of meaning, worthiness, and connectedness (12). The absence of strong social supports is therefore harmful to both the physical and psychological well-being of residents, and can lead to excess mortality risk (13). This is particularly the case for residents with dementia (14). When visitation is restricted or stopped, these interactions are lost. This also negatively affects visitors (family members and friends), disrupting bonds, coping mechanisms, and even their identities (15, 16). Families recognize their role as essential to quality care (17). Indeed, during this pandemic family caregivers have been recognized as the “invisible workforce” that has provided essential care and alleviated strain on health and social care systems (18).

Visiting restrictions may impact most negatively on those who continue to provide personal care to relatives after they institutionalized. Caregivers report difficulty coping with separation after placement (19). Spouses, those providing physical care and those who visit residents daily report the highest levels of anxiety and depression with almost half of visitors at risk of depression (20). These psychological symptoms are often as high as levels experienced prior to admission. Reduced control, personal and cultural expectations and greater worry over perceived decline of the resident may contribute to these findings (20). Few studies have examined the effects of visiting restrictions on caregivers and other visitors of residents. The importance of visiting rituals, particularly on those with cognitive impairment (CI) including dementia is also poorly understood (21). We hypothesized that visitors of residents with CI experience a disproportionally worse impact of visiting restrictions during the COVID-19 lockdown. Give these points, we conducted an online survey to quickly gather information to begin to postulate on the effects of COVID-19 visiting restrictions on measures of perceived loneliness, well-being and caregiver quality of life (QoL) amongst visitors of residents, comparing those with and without cognitive impairment in Ireland.

## Materials and Methods

### Data Collection and Participants

This study is part of Engaging Remotely in Care (ERiC) project (https://www.ucc.ie/en/nursingmidwifery/research/theericprojectengagingremotelyincare/) with the goal of understanding better the impact of public health measures during COVID-19 on families, guardians, and close friends of individuals in RCFs. We developed a novel cross-sectional online survey using Google Docs. Data were collected using convenience sampling. The link to the survey was circulated through university mailing lists via the schools of nursing in colleges across Ireland. Social media accounts of local and regional newspapers were also targeted. Data were collected for 2 weeks up until the 30th of June 2020. Visitors (family members, friends, and legal guardians) of residents currently residing in RCFs in Ireland were eligible to complete the survey. All responses were anonymous and could not be linked back to specific patients. The online instrument was piloted by the research team and amended based on feedback. Informed (online) consent was required prior to respondents completing the questionnaire. Information on the nature of the survey, its purpose and the potential benefits and risks of participation were provided. The survey was entitled “*Impact of public health restrictions on families, guardians, and close friends of residents in Residential Care Facilities*.” Ethical approval was provided in advance after review by the Social Research Ethics Committee (SREC) of University College Cork (UCC).

### Measures

#### Characteristics

A broad range of demographics were obtained from respondents. These included their own age (categorized into: 18–44, 45–54, 55–64, and 65+ years of age), sex, relationship to the resident (close family, friend, or guardian), their own employment status and living arrangements (alone or with others). The clinical status of the resident was also recorded including their approximate length of time in the RCF, location of the RCF unit (geographically by county or city, which were categorized by province, and by urban or rural setting). Respondents were asked whether the resident had CI and if known, whether this represented established dementia and if so, its stage (mild-moderate or severe). Whether the resident was receiving end-of-life care was also asked. The extent of the visitors' caregiving role was assessed by asking about their frequency of visits and the usual purpose of visits (activity based, direct provision of care). Specific questions related to COVID-19 were asked. As well as the perceived impact on communication with RCF staff during the COVID-19 pandemic, visitors satisfaction with care was measured on a Likert scale (from 1 “increased,” 2 “the same,” to 3 “decreased,” dichotomized as “increased/same” or “decreased”) during this time. Resident COVID-19 status (if known) was requested. Subjective reporting of whether they noted changes in the mood, activity of daily living (ADL) function or cognition while participating in phone or other interactions during visiting restrictions were sought. Whether they felt the resident was coping well with these restrictions was also asked.

#### Scales

Specific scales to assess the psychological status of visitors during the COVID-19 visiting restrictions were completed as part of the survey in order to infer their psychological impact. Subjective psychological well-being was scored with the World Health Organization Five Well-being Index (WHO-5) (22). Its structure mirrors the Major Depression Inventory measuring ICD 10 symptoms of depression (22). The raw score is calculated by totaling the responses of five Likert-scale questions exploring the frequency of recent (two-weeks) depressive symptoms (from zero “all of the time” to 5 “none of the time”). Scores range from 0 to 25. Zero represents the worst possible score and hence possible depression and 25 the best possible psychological well-being. A percentage score can be obtained, ranging from 0 to 100%, by multiplying the raw score by four. Loneliness was measured with the University of California, Los Angeles (UCLA) brief loneliness scale (2004 version) (23). This is a 20-item scale measuring the frequency with which an individual feels disconnected from others. Here, we used the first three items (each question was asked as “Thinking of your life as it is now.” with responses rated on a three point Likert scale as “hardly ever,” “some of the time,” and “often”). These were combined to calculate a “loneliness score” from 3 to 9 for each respondent. The lowest possible combined score on this modified version of the scale was 3 (indicating less frequent loneliness) and the highest was 9 (indicating more frequent loneliness). Carer QoL was measured with the Adult Carer QoL (AC-QoL) Questionnaire (24). It is a valid and reliable scale to assess caregivers' perceived challenges and resources (25). Although it has eight subscales, this study only applied one subscale (*Support for Caring*). This subscale measures the extent of support adult carers perceive that they receive, in this case in relation to staff at the RCF, encompassing emotional, practical, and professional support. The subscale includes five questions, each a four-point Likert scale (recording responses from “never” to “always”), giving a possible range of scores from 0 to 15. Higher scores indicate greater QoL; scores of 0–5 indicate a low reported QoL life, and may suggest problems or difficulties.

### Statistical Analysis

Data were analyzed using SPSS V25.0 (Chicago, Illinois, USA) and R version 3.5.0 (2018-04-23)—“Joy in Playing” (26). Numerical data were assessed for normality using the Shapiro Wilk test, Kolmogorov–Smirnov test, and Q–Q plots and all were found to be non-normally distributed. Median and interquartile ranges were therefore reported and compared using the Mann–Whitney *U*-test. Three or more independent samples were compared with the Kruskal–Wallis test. Most data were categorical and frequency distributions (proportions) were compared with Chi-square tests. Linear regression was used to examine the strength of relationship between variables. In order to appreciate if multicollinearity influenced the results of the regression analysis, variance inflation factors (VIFs) were calculated. VIF measure how much the variance of the estimated regression coefficients are inflated compared to when the predictors are not linearly related (27). A generic threshold of ≥10 was applied to assess multi-collinearity (28), scores less than this indicating low risk of multicollinearity.

## Results

### Respondent and Reported Resident Characteristics

In all, 230 responses were received. Of these, 225 were valid and were included in this analysis (i.e., duplicates were removed). Most respondents (91%) identified themselves as immediate family (“*Family who supports the person living in residential care such as spouse, son, daughter, in-law, etc*.”), the remainder as friends or legally appointed representatives. The majority were female (82%). Only 13% were aged ≥65 years; the majority (68%) were aged between 45 and 64 years. Eleven were aged between 75 and 84 years and only one respondent was aged ≥85. Most missing data were found for the “diagnostic condition list,” with only 202 responses recorded for “any history of CI.” A summary of responses from these are presented in Table 1. Most (80%, 162/202), identified that their resident had CI with 45% self-reporting this to be severe dementia. In all, 10% stated that the resident was receiving end-of-life care. Most nursing homes were in rural or suburban locations rather than urban; most were in the east and south of the country, where the two largest cities are located, Dublin and Cork, respectively. Half of these respondents indicated that prior to restrictions that they “always” or “usually” engaged in activities with residents when visiting and one-fifth that they “always” or “usually” engaged in personal care with the resident. A higher proportion of those reporting that their resident has CI responded that they visited more frequently (*p* = 0.01) and that they “always” or “usually” engaged in personal care (25%) compared to those not reporting CI (13%), although this did not reach statistical significance (*p* = 0.097).

**Table 1:** Summary of survey responses including a comparison between respondents of residents with and without cognitive impairment.

**Variable**	All respondents^*^ (*n* = 202)	**Residents living with known cognitive impairment (*n* = 162)**	**Residents without known cognitive impairment (*n* = 40)**	**Significance testing (*p*-value)**
**Demographics (reported by respondents)**				
**Age**				
18–44 years	39 (19%)	33 (20%)	6 (15%)	0.598
45–54 years	76 (38%)	63 (39%)	13 (33%)	
55–64 years	59 (29%)	45 (28%)	14 (35%)	
65+ years	28 (14%)	21 (13%)	7 (18%)	
Sex (% female)	165 (82%)	135 (83%)	30 (75%)	0.222
Relationship to resident (% close family)	184 (91%)	146 (90%)	38 (95%)	0.332
Employed	136 (67%)	112 (69%)	24 (60%)	0.270
Living arrangement (% living alone)	38 (19%)	31 (19%)	7 (18%)	0.813
**Resident characteristics**				
**(reported by respondents)**				
Institutionalized for at least a year (%)	141 (70%)	115 (71%)	26 (65%)	0.460
Resident with severe dementia (%)	73 (36%)	73 (45%)	0 (0%)	N/A
Resident receiving end-of-life care	19 (10%)	14 (9%)	5 (13%)	0.469
**Location of nursing home**				
Northwest (Connacht/Ulster)	29 (14%)	23 (14%)	6 (15%)	0.908
East (Leinster)	87 (43%)	71 (44%)	16 (40%)	
South (Munster)	86 (43%)	68 (42%)	18 (45%)	
Urban vs. Rural (% urban)	78 (39%)	60 (37%)	18 (45%)	0.354
**Contact time (usual frequency of visits)**				
At least twice a week	122 (60%)	102 (63%)	20 (50%)	0.010
Weekly to fortnightly	69 (34%)	55 (34%)	14 (35%)	
Several time a year or less	11 (5%)	5 (3%)	6 (15%)	
**Visitor role**				
Provide care (% who always/usually do)	45 (22%)	40 (25%)	5 (13%)	0.097
Do activities with resident (% who always/usually do)	101 (50%)	83 (51%)	18 (45%)	0.480
**Impact of COVID-19 on visitor and resident (perceived/reported by respondents)**				
Resident positive for COVID-19 (% positive)	18 (9%)	14 (9%)	4 (10%)	0.751
Impact of visit restrictions (visitor)				
Significant impact on communication	77 (38%)	59 (36%)	18 (45%)	0.317
Decreased satisfaction with care	55 (27%)	46 (28%)	9 (23%)	0.554
**Impact of visit restrictions (resident)**				
**Resident coping well**				
Yes	64 (32%)	48 (30%)	16 (40%)	0.315
Don't know	39 (19%)	34 (21%)	5 (13%)	
No	99 (49%)	80 (49%)	19 (48%)	
Change in mood (% Yes)	109 (54%)	83 (51%)	26 (65%)	0.055
Change in functioning (% Yes)	86 (43%)	69 (43%)	17 (43%)	0.122
Change in memory (% Yes)	104 (51%)	87 (54%)	17 (43%)	0.002

* *This analysis only included those who responded to whether their resident was known or not known to be living cognitive impairment; Note 225 valid answers were received but 23 were missing data for cognitive impairment. N/A, Not applicable*.

### Perceived Impact of COVID-19

The next section of the survey assessed the perceived impact of the COVID-19 pandemic on respondents and the resident as perceived by respondents. This analysis focuses on the 202 responses where the presence or absence of CI was indicated. Eighteen of those with a response to the question on CI (9%) answered that their resident had been diagnosed with COVID-19. In all, 38% indicated that visiting restrictions had a significant negative impact on communication with RCF staff and 27% reported decreased satisfaction with care. Visitors who reported lower satisfaction with care had statistically significantly lower self-reported well-being, a median WHO-5 Well-being Index score percentage score of 44 vs. 60%, (*p* = 0.01). Similarly, those reporting lower levels of satisfaction with the support offered by RCF staff (based on the item from the Adult Carer QoL Questionnaire) had significantly lower WHO-5 scores (*p* = 0.002).

Most and almost half of respondents (49%) reported that their resident was not coping well with restrictions. One in five did not know and one-third reported that there were coping. Half reported that their resident displayed a negative change (reduction) in mood, ADL function and memory during the pandemic. Comparing residents with and without reported CI, those living with CI were noted by visitors to have statistically significantly greater reductions in memory during the period of restrictions, 54 vs. 43% (*p* = 0.002). Examining the scales to infer the psychological impact of restrictions on respondents showed that median (interquartile) UCLA brief loneliness scale scores were 4/9 (±3), WHO-5 well-being scores were 56/100 (±36), and AC-QoL scores were 9/15 (±6), see Table 2. In all, 72/162 (44%) reported WHO-5 scores below 50%.

**Table 2:** Outcome measures for survey respondents assessing the psychological status of visiting restrictions during COVID-19 pandemic 2020.

**Outcome measure**	All residents^*^ (*n* = 202)	**Residents with cognitive impairment (*n* = 162)**	**Residents without cognitive impairment (*n* = 40)**	***p*-value**
**WHO-5 Well-being Index score**				
Raw score (Median and IQR)	15 (10–19)	14 (9–19)	19 (11.5–20)	0.006
**WHO-5 Well-being index score**				
Percentage score (Median and IQR)	60 (40–76)	56 (36–76)	76 (46–80)	
**UCLA brief loneliness scale** (modified version) (Median and IQR)	4 (3–6)	5 (3–6)	3.5 (3–6)	0.114
**AC-QoL Questionnaire** (support for caring subscale) (Median and IQR)	10 (7–13)	10 (7–12)	10 (6.5–14)	0.306
**Family perception of care scale (Median and IQR)**				
Total score	23 (18–28)	23.5 (19–29)	22 (15–27.5)	0.183
Resident care subscale	15 (11–18)	15 (12–19)	14 (8.5–17)	0.138
Communication subscale	8 (11–6)	8 (6–11)	7.5 (6–10.5)	0.558

* *Two-hundred and twenty-five answered survey but 23 are missing data for cognitive impairment*.

*AC-QoL, Adult Carer Quality of Life Questionnaire; WHO, World Health Organization; N/A, Not applicable*.

On the AC-QoL, ~one-fifth (17%) of respondents scored 0–5/15, indicating that support they received from RCF staff during this period was perceived to be poor. This suggests low self-reported QoL. Visitors of those with CI reported statistically significantly lower well-being scores over the past two weeks (56 vs. 76%, respectively, *p* = 0.006) but no difference in loneliness scores (*p* = 0.114) or carer QoL scores (*p* = 0.305). Linear regression modeling, showed that reported CI (*p* = 0.04) was an independent predictors of WHO-5 scores, after adjusting for age, sex, dementia stage (proportion with reported advanced dementia), perceived professional support provided by RCF staff (item taken from the Adult Carer QoL Questionnaire) and satisfaction with care (proportion reporting decreased satisfaction), see Table 3. Examining only those visitors reporting reduced satisfaction with care (*n* = 55), found no difference in WHO-5 scores after adjusting for age, sex, CI, and the presence/absence of perceived support from RCF staff. All VIFs for individual variables included in the regression models were marked lower than 10, indicating a low risk of collinearity.

**Table 3:** Linear regression model showing the association between variables and WHO Well-being Index scores (range 0–100).

**Variable**	**Estimate**	**Standard Error (S)**	***p* = X**
**Age** (category)	0.22	2.26	0.92
**Sex** (female)	−8.77	4.91	0.08
**Location of RCF** (urban)	0.05	0.28	0.86
**Staff support** (Item from Adult Carer QoL Questionnaire; % satisfied “Some of the time” or “Never”)	−5.11	5.18	0.33
**Satisfaction with care** (% reduced)	−7.92	5.30	0.14
**Cognitive Impairment** (impaired)	11.46	5.46	0.04^*^
**Dementia stage** (advanced)	−0.006	4.73	0.99

*N = 202; Adjusted r square = 0.08*.

*QoL, Quality of Life; RCF, Residential Care Facility*.

* *Statistically significant (0.038)*.

## Discussion

This study, a national survey of family, friends, and guardians of residents in RCF in Ireland, conducted during the COVID-19 pandemic, found that a large proportion of respondents reported recent low well-being as well as feeling lonely and isolated. Almost a fifth reported that support for their role as caregivers from staff in RCFs was poor and that they had a low self-reported QoL as a result. Approximately one-third of respondents remarked that they were dissatisfied with care and that restrictions had impacted on the care of residents. Those reporting that their satisfaction with care received by their resident and with the support provided by RCF staff to them (taking the “happiness with professional support” item from Adult Carer QoL Questionnaire) were reduced during the lockdown were statistically significantly more likely to report lower well-being. Most perceived that residents were not coping well during this period. This may have impacted on their own feelings and perceptions of well-being, explaining the relatively low median WHO-5 well-being index scores and large proportion (44%) scoring <50%. This is not unexpected given that pandemics are associated with a range of negative psychological effects (29).

This study compared the responses of visitors reporting that their resident was living with CI with those that did not. Whether the cognitive status of residents may have influenced self-reporting of a range of psychological measures of mood (depression), loneliness, and QoL was examined. The results for scores on the WHO-5 here suggest that respondents of residents with CI have statistically significantly poorer well-being scores and were more likely to be depressed. Linear regression showed that this remained significant after adjusting for potential confounders including the stage of dementia. The WHO-5, a short questionnaire consisting of five simple and non-invasive questions examining subjective well-being of the respondents, is a validated and accurate screening tool for depression. It is widely-used as an outcome measure in clinical trials across a broad range of scientific fields (22). Differences between those with and without CI may reflect different tensions and concerns specific to those visitors and the loss of their caring role during visiting restrictions. That families of those with dementia play a particularly active role in visiting residents with dementia supports this (30).

Of particular concern is that the majority of respondents who were in contact with residents during this period noted a decline in the mood, ADLs, and cognition (memory). This was significantly different (higher proportion) for those with CI with over half of these responding in the affirmative. This would be expected given the importance contact with family and friends has for residents with CI, particularly their role in supporting activities including cognitively stimulating activities (31) and in maintaining resident QoL (32). It is probable that the restriction of visits for a prolonged period is directly attributable to this decline, albeit this is a reported and unsubstantiated deterioration that may reflect respondents own concerns with the residents care.

### Strengths, Limitations, and Next Steps

This study has a number of strengths and limitations affecting the interpretation of the results. Convenience sampling was used, potentially limiting the representativeness of the final sample obtained. Responses were predominantly from the provinces of Munster and Leinster (the two largest population centers), particularly from Cork in Munster where UCC is based. Few responses were from the West and North of the country. This indicates possible selection bias (under-coverage). It is likely that only the most motivated and computer literate respondents completed this online survey, introducing voluntary response and non-response bias. Other approaches to gathering data and more representative sampling should therefore be considered a priority. Most respondents (67%) were in the 45–64 year old age group (often children of residents), further reducing the generalizability of the findings. However, this represents the key age cohort for caregivers in Ireland with most aged between 45 and 64 years (33). A large majority of respondents to this survey were female, again potentially reducing the generalizability of the study, although this mirrors the demographic make-up of Irish carers (33), and higher numbers of female visitors to RCFs are reported in many studies, e.g., the Netherlands (34). Further, proportions were not significantly different between those with and without known CI. Most (91%) identified themselves as close family who usually support the resident. Given that these have an important role in supporting the care of people in RCF and are themselves more prone to anxiety and depression related to the institutionalization of their family member (20), the psychological impact of COVID-19 restrictions may be reflective of the true impact on families who usually support residents. The small sample size is a weakness of the study, representing only ~1% of residents in RCF in Ireland; there are ~22,500 residents aged over 65 years in nursing home care (35). This also limits the representativeness and generalizability of findings. However, sample size, as well as the design, should be informed by the purpose of a mental health survey (36). In this case, it was to quickly gather information to generate ideas, suggesting that rapid, low-cost convenience sampling may be acceptable (36). The need is engendered by the paucity of data on the psychological impact of the COVID-19 pandemic on visitors to RCFs. Larger samples may not necessarily overcome these biases, hence having a reasonably representative samples of visitors as is inferred by the demographics of this sample, is important. Nevertheless, the authors emphasize that associations found in this research may not reflect the true impact of COVID-19 on the target sample and causality cannot be inferred.

It is unclear how many residents are represented by the survey as different family members of the same resident could in theory have responded to the survey. While this could not be determined, it was possible to identify if the same individual attempted the survey a second time. All responses from the same IP address were removed. Another limitation is that there were some randomly distributed missing data. This can lead to bias and reduced precision when analyzing patient-reported outcomes (37). Surveys are prone to having missing data although in this case, the number of missing values was low. To address this, as most data were categorical, rather than imputing data, missing values were automatically removed (38). Further, the design of the survey minimized missing data by making key questions mandatory in order to progress to the end of the questionnaire.

As all responses were anonymous, the accuracy of responses could not be verified. It was therefore not possible to confirm whether information on diagnoses reported (e.g., the presence of dementia and its stage) were accurate and correctly classified. Such responses are prone to reporting bias and error. Nevertheless, the proportion of residents with reported CI in this study at 80%, is similar to the suggested true prevalence of dementia in nursing homes in Ireland, which although frequently under-diagnosed may be as high as 90% (39). Similarly, no data were available about the nursing homes included in the study. A follow-on study of both residents and of staff in the nursing homes and their view of the impact of lockdown restrictions on RCFs is planned as part of the ERIC project. The design of the study also limits the interpretation of the results. Specifically, as the study was cross-sectional, it was not possible to ascertain the baseline scores of the scales used to measure the psychological impact of visiting restrictions during the COVID-19 pandemic. As no measures were obtained prior to the lockdown, it is also impossible to determine whether these changed as a result of the lockdown. All responses reflected the well-being, QoL, and loneliness in a moment in time (recent weeks), though it was not possible to ascertain if the scores truly reflect the impact of COVID-19. While it is possible that having a relative in residential care with CI, heightened the negative psychological impacts of COVID-19, the cross-sectional nature of this survey means that causation cannot be inferred. This said, visitors of those with CI, are known to experience lower well-being at baseline and during periods of crisis including at the end-of-life (40). Similarly, CI and its severity are known to increase carer stress and burden (41). Further, this survey was conducted almost 4 months into the ongoing pandemic and asked specifically about COVID-19 and their experiences as well as the perceived experiences of their relative/friend during this period.

Finally, two out of three of the scales used to assess the psychological status of visitors during the COVID-19 RCF visiting restrictions were truncated, i.e., these were mostly sub-scales or sub-sections of the original scales with reduced reliability. This also reduces the generalizability and comparability of the findings. The decision to use these modified or subscale versions was made to minimize the length and complexity of the questionnaire, particularly given the broad target sample (ranging from younger caregivers/relatives to older spouses). This was largely successful given that the vast majority of questionnaires were completed fully with a relative paucity of missing data. Further, there is a need to combine existing scales as none have been specially designed and tested against the backdrop of a pandemic of this nature. Nevertheless, these are widely-used scales and their subscales are often used as stand-alone assessments of psychological well-being in studies. The WHO-5 for example, is validated as a screening tool with high sensitivity for both major and minor depression. It is shorter than the GDS-15 and is superior to the GDS-4 (42). Reducing the number of items was also important to attempt to limit the possibility of multicollinearity. As collinearity among covariates is an almost inevitable problem when analyzing survey data, VIFs were calculated taking a generic cut-off of ≥10 to assess this (28). VIFs are robust and account for complex design features (27). In these analyses, VIFs calculated for variables in the regression models indicated a low probability of collinearity.

Although visiting restrictions to RCFs in Ireland have begun to ease over recent weeks, the requirement to socially distance, wear face covering and limit visits to RCFs (both in duration and frequency) is likely to continue as the pandemic keeps up pace. This reinforces the need to develop solutions to overcome these restrictions (36) and improve communication and remote contact between visitors, residents and staff in RCF (43). These findings, limited in size and to a single country, should be examined in other settings and countries. Hence, research is now required to understand whether reduced well-being among respondents of residents reported to be living with CI is due to disrupted caregiving roles resulting from the restrictions imposed during this pandemic. The loss of this role and its associated meaning could account for such changes (15, 16).

Future research should likewise examine not only the impact of COVID-19 restrictions in RCFs on visitors but also on residents themselves, particularly given the pivotal role these visitors and their visits play in providing support for activities and the personal care of residents. Studying measures to mitigate the psychological impact is also required. To date, little research has been conducted into this with anecdotal evidence suggesting that social isolation during the pandemic is having seriously harmful consequences on residents including increased anxiety, depression, loneliness, and worsening dementia (44, 45). Given the pressing need to understand the prevalence of the psychological impact of COVID-19 on both residents and families, future surveys should therefore use rigorous methods that sample from the whole population (36). Qualitative studies would help shed light on the impacts.

## Conclusions

In summary, this pragmatic hypothesis-generating study is the first to our knowledge to examine how visiting restrictions to RCFs during COVID-19 may have impacted on the psychological status of a variety of visitors but predominantly close family. The results indicate that many nursing home visitors are experiencing low psychological and emotional well-being during this pandemic. Well-being was significantly lower for those reporting that the resident they are connected with has CI. It may be that visitors and carers of those with CI in RCFs are experiencing lower well-being than those without known CI but limitations in the study design limit our ability to confirm this. We suggest that this may be related to visiting restrictions themselves, although further research is also required to evaluate this and the role staff working in RCFs can have in supporting visitors to mitigate reduced well-being during this pandemic. If confirmed there will be a need to identify measures to address their impact over a prolonged period, given the current lack of adequate treatments or a vaccine. The impact on residents and staff must also be investigated.

## Data Availability Statement

The raw data supporting the conclusions of this article will be made available by the authors, without undue reservation.

## Ethics Statement

This study was reviewed by the Social Research Ethics Committee of University College Cork. The patients/participants provided their written informed consent to participate in this study.

## Author Contributions

RO'C: design, statistical analysis, writing, and revising manuscript. MM, CD, CB, CK, and SF: concept, design, and data collection. MO'D: data management and statistical analysis. IH: concept, design, concept, and supervision. NC: concept, design, data collection, writing, and revising manuscript.

## Conflict of Interest

The authors declare that the research was conducted in the absence of any commercial or financial relationships that could be construed as a potential conflict of interest.
